# Visualization of Real‐Time Esophageal Location Using Intracardiac Echocardiography on a Three‐Dimensional Mapping System: Comparison of Esophageal Location Using Preoperative Computed Tomography and Investigation of Predictors for Esophageal Movement During Catheter Ablation

**DOI:** 10.1111/jce.70096

**Published:** 2025-09-19

**Authors:** Koji Sudo, Kenji Kuroki, Chisa Asahina, Maoko Atsumi, Kazuya Nakagawa, Tetsuya Asakawa, Tomoaki Hasegawa, Kazutaka Aonuma, Akira Sato

**Affiliations:** ^1^ Department of Cardiovascular Medicine University of Yamanashi Chuo Japan; ^2^ Department of Cardiology Yamanashi Kosei Hospital Yamanashi Japan; ^3^ Department of Cardiology Mito Saiseikai General Hospital Mito Japan

**Keywords:** atrial fibrillation, esophageal injury, high‐power short‐duration ablation, intracardiac echocardiography, pulmonary vein isolation, radiofrequency catheter ablation

## Abstract

**Introduction:**

Pulmonary vein isolation (PVI) using radiofrequency catheter ablation is an effective treatment for atrial fibrillation; however, esophageal‐related complications remain a concern. The first objective of this study was to compare the feasibility of two techniques for visualizing real‐time esophageal images: intracardiac echocardiography (ICE‐Eso) and preoperative computed tomography (CT‐Eso). The second objective was to clarify the predictors of esophageal movement on the day of catheter ablation.

**Methods and Results:**

Eighty consecutive patients were included in this study. The esophageal location was measured at the centerline of each image on three equally separated imaging sections (upper, middle, and lower sites). Esophageal locations detected on ICE‐Eso and CT‐Eso were compared with those on contrast esophagography. We also investigated predictors of esophageal movement. A significant difference was found between the two distances in all three sections: upper site (ICE‐Eso: 2.5 [interquartile range (IQR) 1.4–3.6] mm vs. CT‐Eso: 5.2 [IQR 3.4–7.6] mm, *p* < 0.001), middle site (ICE‐Eso: 2.7 [IQR 1.3–4.3] mm vs. CT‐Eso: 5.4 [IQR 3.2–8.3] mm, *p* < 0.001), and lower site (ICE‐Eso: 2.8 [IQR 1.2–5.2] mm vs. CT‐Eso: 5.8 [IQR 3.1–10.3] mm, *p* < 0.001). Multivariate analysis revealed that eating a meal on the morning on the day of catheter ablation (non‐fasting) was a predictor of esophageal movement. One patient (1.2%) experienced gastric hypomotility, which resolved completely with medical treatment.

**Conclusion:**

The results showed that ICE‐Eso provided real‐time, accurate esophageal location compared to CT‐Eso. Therefore, ICE‐Eso‐guided PVI on the left atrial posterior wall near the esophagus may be a safe method. Additionally, non‐fasting on the day of catheter ablation could help to predict esophageal movement.

## Graphical abstract

Esophageal imaging using intracardiac echocardiography (ICE‐Eso) showed a more accurate real‐time esophageal location than preoperative computed tomography (CT‐Eso). Non‐fasting on the day of catheter ablation (CA) may be a predictor of esophageal movement on the day of CA. Therefore, ICE‐Eso‐guided pulmonary vein isolation is a safe method for non‐fasting patients.

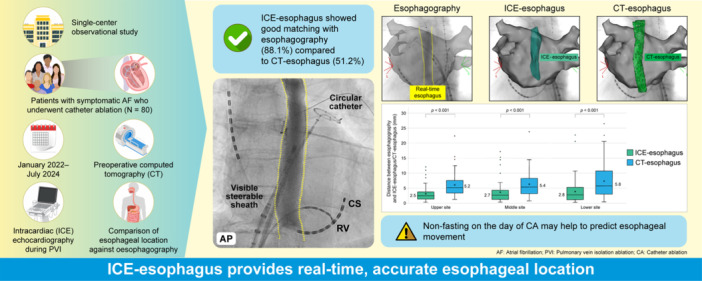

**Abbreviations**
3D: Three‐dimensional
AF: atrial fibrillation
AI: ablation index
CA: catheter ablation
CF: contact force
CT: computed tomography
Eso: esophageal
HPSD: high‐power short‐duration
ICE: intracardiac echocardiography
LA: left atrial
LAPW: left atrium posterior wall
PVI: pulmonary vein isolation
RF: radiofrequency

## 1 Introduction

Pulmonary vein isolation (PVI) is an effective treatment for patients with atrial fibrillation (AF). When performing radiofrequency (RF) catheter ablation (CA) near the left atrial (LA) posterior wall (LAPW), the risk of thermal injury to the adjacent esophagus increases substantially [1, 2]. For example, one complication is atrio‐esophageal fistula, a rare but life‐threatening complication associated with AF treatment [3]. However, one study suggested that RF application using a high‐power, short‐duration (HPSD) ablation protocol might reduce esophageal injury [4].

In a study by Halbfass et al., the only risk factor associated with the occurrence of esophageal injury or ulcer formation was intra‐esophageal temperature rise [5], but esophageal temperature probes are effective tools to resolve this issue [6, 7, 8]. However, some clinical studies have reported that these probes may contribute to a rise in esophageal temperature and increase the risk of injury. Although mechanical esophageal deviation offers a safer method for monitoring esophageal damage [9, 10], there is still no guarantee that esophageal‐related complications will not occur.

Intracardiac echocardiography (ICE) combined with a three‐dimensional (3D) mapping system is an important real‐time imaging modality for elucidating the anatomy of the LA during fluoroless CA procedures [11, 12, 13]. Hayashi et al. recently reported the utility of ICE introduced into the LA for validating esophageal visualization methods and analyzing the thickness of the LAPW adjacent to the esophagus. These authors also demonstrated an effective ablation method that used a low‐power setting (25 W) guided by an ablation index (AI) with a relatively low target value of 260 for the esophageal location [14]. In this study, we aimed to compare the accurate real‐time esophageal location between ICE (ICE‐Eso) and preoperative computed tomography (CT‐Eso). We also examined whether the HPSD ablation protocol using ICE‐Eso on the LAPW near the esophagus was a safe and efficient method. In addition, we investigated how the timing of preoperative meals impacted the prediction of esophageal movement.

## 2 Methods

### 2.1 Study Design and Participants

This single‐center observational study evaluated 80 consecutive patients who underwent RFCA for AF between January 2022 and July 2024 at Yamanashi University Hospital. Patients with symptomatic, drug‐refractory AF were enrolled. All patients underwent preoperative CT within the 3 weeks before the procedure to exclude intracardiac thrombi and confirm pulmonary vein anatomy. In patients with a history of cerebral infarction, intracardiac thrombi were ruled out using transesophageal echocardiography.

The inclusion criteria were patients aged > 18 years who were scheduled for an RFCA procedure for symptomatic drug‐refractory AF. Paroxysmal AF was defined as AF lasting for < 7 days. The exclusion criteria were patients who had an LA diameter > 60 mm on transthoracic echocardiography and those who had severe valvular heart disease, worsening heart failure, or intracardiac thrombus. In addition, two patients were excluded because they had requested deep sedation during CA.

### 2.2 Definitions of Fasting and Non‐Fasting

Fasting time was calculated as the time from the most recent solid food intake to the time of admission to the operating room. Fasting itself was defined as a minimum of 6 h without solid food and at least 2 h without liquids before the scheduled start of the CA. Non‐fasting was defined as the intake of solid food within the 6 h before transfer to the operating room. Medication intake was not included in the fasting criteria.

### 2.3 Ethical Approval

This study was conducted using an opt‐out method. The requirement for obtaining individual informed consent was waived because only deidentified data were used. Instead, information regarding the study was disclosed on the website of our institution, where patients had the option to decline participation. This study was approved by the Institutional Review Broad of the University of Yamanashi (Approval no. 2773).

### 2.4 Ablation Procedure

All procedures were performed using dexmedetomidine for minimal to moderate sedation and fentanyl as an intravenous analgesic. Following venous puncture, 5000 units of heparin were administered. Femoral arterial access was performed regularly for continuous monitoring of arterial pressure. A 6‐Fr, 20‐pole, 3‐site mapping catheter (BeeAT; Japan Lifeline, Tokyo, Japan) was placed in the coronary sinus via the right jugular vein for pacing, recording, and internal cardioversion. After transseptal puncture with a needle catheter (RF needle; Japan Lifeline, Tokyo, Japan) guided by fluoroscopic imaging and real‐time ICE (CARTO SOUNDSTAR ultrasound catheter, Biosense Webster, Diamond Bar, CA), 3000 units of heparin were administered, followed by continuous heparin infusion to maintain an activated clotting time of 350–400 s throughout the CA procedure. A visible steerable sheath (VIZIGO, Biosense Webster) and a long sheath (SL‐0, Abbott, St Paul, MN, USA) were introduced into the LA via the same transseptal puncture site and continuously flushed with heparinized saline. A high‐resolution multipolar mapping catheter (Pentaray or Octaray, Biosense Webster) was inserted and integrated into the 3D mapping system for LA geometry construction and voltage mapping.

### 2.5 Esophageal Images Using ICE, Preoperative CT, and Contrast Esophagography

**Figure 1:**
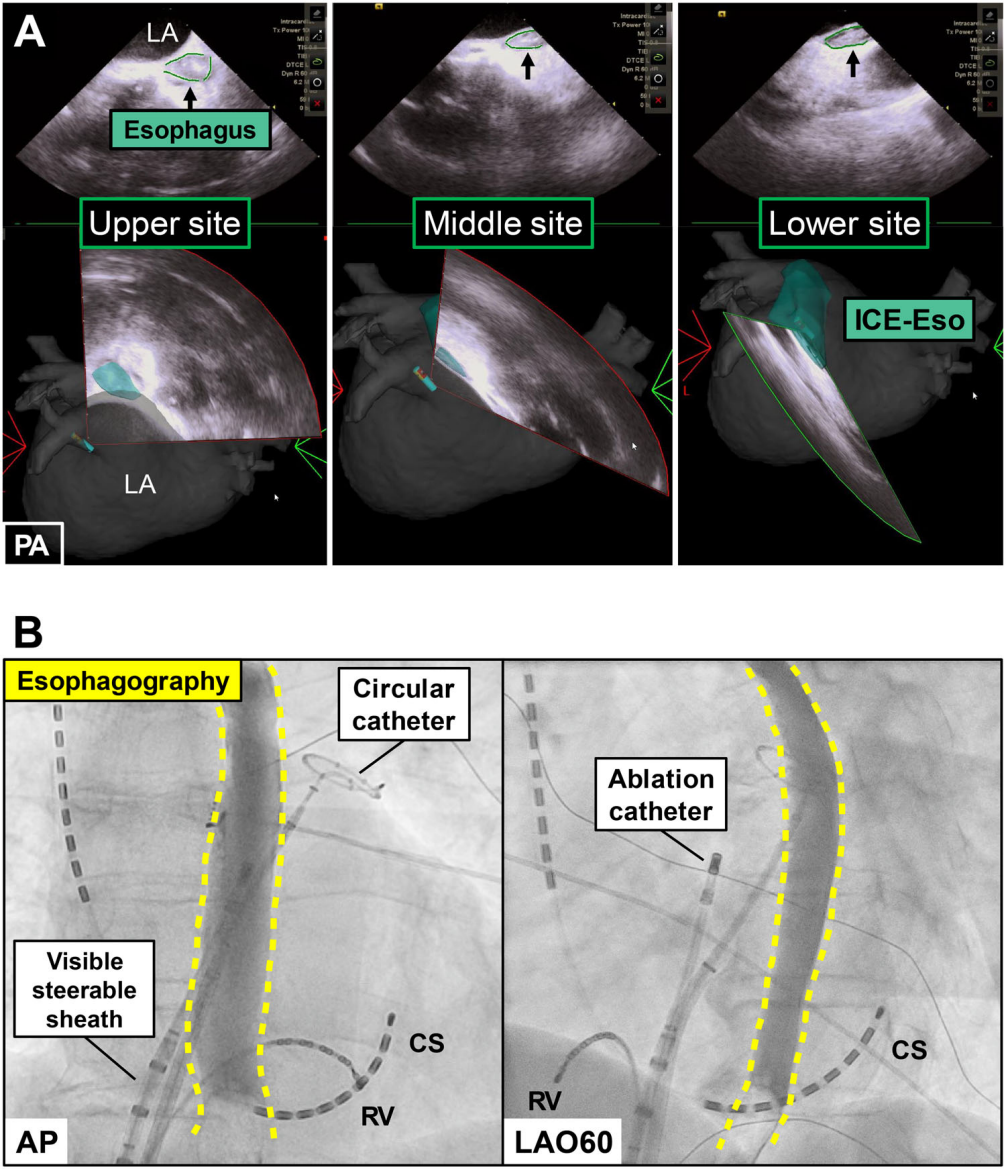
Esophageal Imaging using Intracardiac Echocardiography (ICE‐Eso) and Contrast Esophagography. Panel A: The esophagus using intracardiac echocardiography is shown on three sections in the *green area* (*Left panel*: Upper site, *Middle panel*: Middle site, *Right panel*: Lower site). Panel B: Contrast esophagography using gastrografin shows real‐time esophageal location (*yellow dotted line*) in the anterior‐posterior view (*left panel*) and the left anterior oblique view (*right panel*).

Before electro‐anatomical mapping, the ICE catheter was inserted into the LA via the long sheath and positioned for optimal visualization of the esophageal anatomy. The ICE imaging was visualized on the 3D mapping system to ensure that the echo beam emanated from the LAPW side. The mucosa of the esophagus was outlined with high‐brightness imaging, whereas the periphery of the esophagus was depicted as a low‐echoic area and trace. Multiple ICE slices were required to create a clearer depiction of the esophageal mucosa and encompass the RF ablation sites for PVI, which spanned the roof of the superior pulmonary vein (PV) (upper site) to the bottom of the inferior PV (lower site) (Figure 1A).

**Figure 2:**
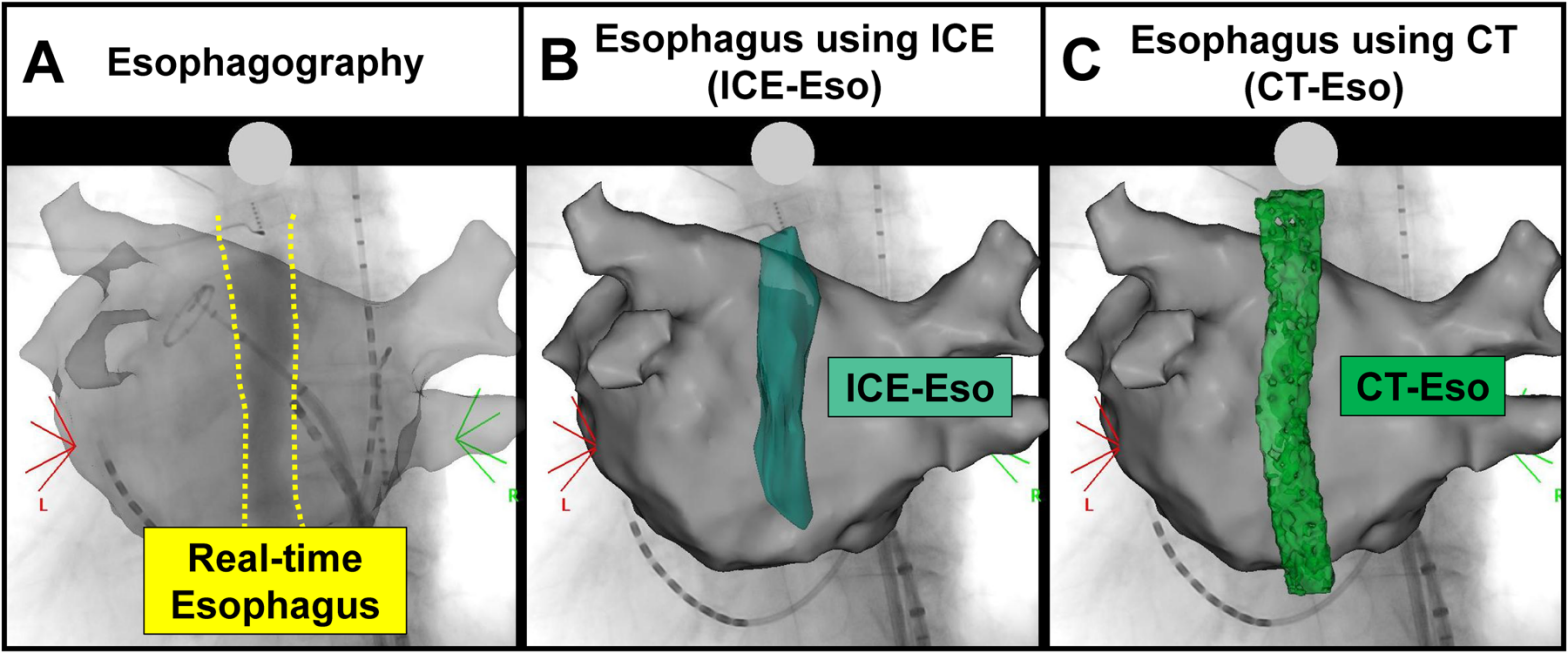
Combination Multimodality Imaging on Three‐Dimensional Mapping System. Multimodality imaging combined with fluoroscopy and CARTO3 system using CARTOUNIVIU module. (Panel A: Contrast esophagography; Panel B: Intracardiac echocardiography; Panel C: Computed tomography.)

After creating the ICE‐Eso image, we performed electro‐anatomical mapping using a high‐resolution multipolar mapping catheter. Subsequently, a circular catheter (EPstar Libero, Japan Lifeline, Tokyo, Japan) was placed at the left superior PV, and contrast esophagography was performed to confirm the real‐time esophageal location. While awake, the patients swallowed 5–10 mL of gastrografin, a bitter‐flavored, water‐soluble contrast medium. During ingestion, fluoroscopic images were recorded in anteroposterior and left anterior oblique views (Figure 1B). Contrast esophagography was integrated into the CARTO3 system (Biosense Webster) using the CARTOUNIVU module (Biosense Webster) (Figure 2A), which corresponded to the ICE‐Eso (Figure 2B) and preoperative CT‐Eso (Figure 2C).

**Figure 3:**
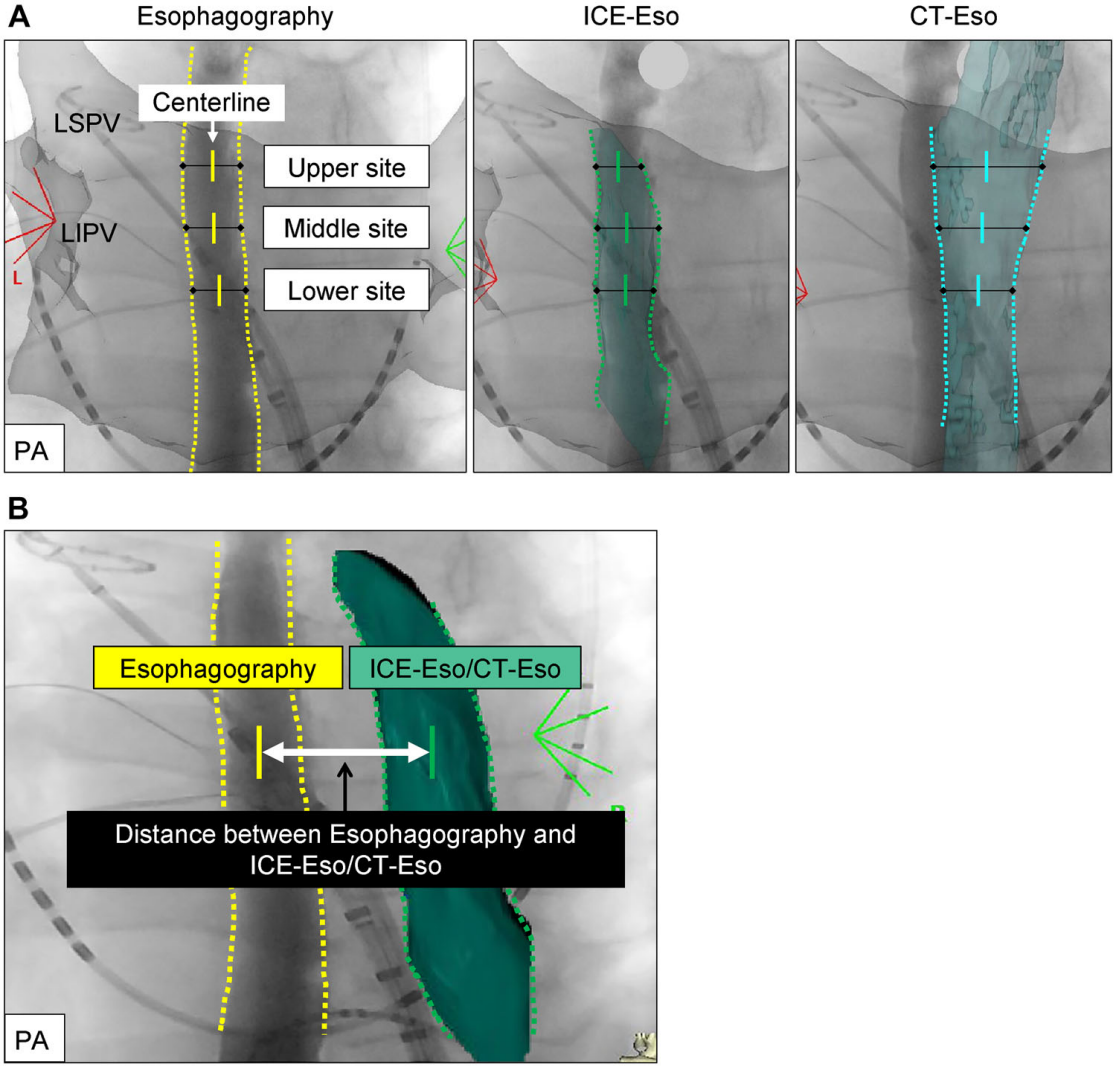
Comparison of Distance Between ICE‐Eso and Contrast Esophagography and Between CT‐Eso and Contrast Esophagography. Panel A: Definition of centerline in each imaging (*left panel*: Contrast esophagography; *middle panel*: ICE‐Eso; *right panel*: CT‐Eso). Panel B: Method of distance measurement between contrast esophagography and ICE‐Eso/CT‐Eso using the centerline of each image.

Measurements were taken at three sections of the esophagus: an upper (left superior PV level), middle (left PV carina level), and lower site (left inferior PV level) (Figure 3A). Esophageal locations detected on ICE‐Eso and CT‐Eso were compared with those on contrast esophagography by measuring the distances between the centerlines on each imaging modality (Figure 3B). One independent physician assessed the distance between esophagography and ICE‐Eso/CT‐Eso. We used information from previous reports [15, 16] to define good matching as < 5.0 mm between the centerline of the esophagography image and that of ICE‐Eso or CT‐Eso in at least two of three sections.

### 2.6 Radiofrequency Catheter Ablation Protocoll

Point‐by‐point RF ablation was performed using an irrigated‐tip with a contact force (CF)‐sensing ablation catheter (Thermocool SmartTouch Surround Flow catheter, Biosense Webster) guided by the 3D mapping system. During PVI, we placed a circular diagnostic catheter in the left or right superior PV to confirm entrance block and PVI. Fentanyl boluses were administered intravenously before RF application.

The RF energy setting was 40 W and the target AI was set at 450 on the LA anterior wall. In line with a previous prospective study, the target AI was set to 400 on the LAPW nonadjacent to the esophagus at each ablation point [17]. Using the HPSD ablation protocol, RF was applied at 50 W for 5 s to the LAPW near the ICE‐Eso. The CF was maintained at 5–15 g at all ablation points, and the lesion tag size (VisiTag, Biosense Webster) was 6 mm in diameter (Supplemental Figure 1).

After PVI, a high‐resolution multipolar mapping catheter was inserted into the left and right PVs to confirm that all PV potentials were either abolished or dissociated from atrial activity. A bidirectional block was documented. Cavotricuspid isthmus ablation was performed at the discretion of the physician. Superior vena cava isolation was performed as an additional ablation if the myocardial sleeve was sufficiently long. For patients who underwent multiple sessions, additional ablations such as non‐PV foci ablation, LAPW isolation, and low‐voltage area ablation were performed.

### 2.7 Statistical Analysis

Continuous variables are expressed as mean ± standard deviation or median with interquartile range [IQR: 25%, 75% percentiles]. Categorical variables are expressed as numbers and percentages. The Mann‐Whitney *U* test was used for between group comparisons of continuous variables. Proportions were analyzed using the *χ* ^2^ test. Statistical significance was set at *p* < 0.05. The Kruskal–Wallis rank sum test and Bonferroni's post hoc test were used for the comparisons of esophageal width among esophagography, ICE‐Eso, and CT‐Eso. To evaluate predictors for esophageal movement compared to preoperative CT imaging, univariate logistic regression analysis was performed to calculate odds ratios and 95% confidence intervals. Multivariate logistic regression analyses were also performed using variables with *p* < 0.20 in the univariate analysis. All analyses were performed using EZR software (Saitama Medical Center, Jichi Medical University, Saitama, Japan), a graphical user interface for R (The R Foundation for Statistical Computing, Vienna, Austria).

## 3 Results

### 3.1 Patient Population

A total of 80 patients were enrolled in this study, of which 54 were males (67.5%). The mean age of the overall group was 70.5 ± 9.8 years. Among the participants, 36 patients (45%) had paroxysmal AF. The CHA_2_DS_2_‐VASc score was 2.7 ± 1.3 points. The mean LA diameter, LA volume index, and left ventricular ejection fraction on transthoracic echocardiography were 42.9 ± 7.7 mm, 43.1 ± 16.1 mL, and 60.6 ± 11.8%, respectively. In addition, 59 patients (73.7%) had fasted on the day of the CA. Table 1 shows the baseline patient characteristics.

**Table 1:** Baseline patient characteristics.

	*n* = 80
Age, year	70.5 ± 9.8
Gender (male/female), *n*	54/27
BMI, kg/m^2^	24.3 ± 4.2
AF type	
Paroxysmal, *n*	36 (45)
Non‐paroxysmal, *n*	44 (55)
Ablation type	
Initial ablation, *n*	76 (95)
Redo ablation, *n*	5 (6.2)
Hypertension, *n*	44 (55)
Diabetes mellitus, *n*	17 (21.2)
Congestive heart failure, *n*	24 (30)
History of stroke/TIA, *n*	8 (10)
CHA_2_DS_2_‐VASc score	2.7 ± 1.3
LVEF, %	60.6 ± 11.8
LA diameter, mm	42.9 ± 7.7
LA volume index	43.1 ± 16.1
BNP, pg/mL	148.7 [40.6–169.7]
Anticoagulation therapy	
Warfarin, *n*	5 (6.3)
DOAC, *n*	75 (93.7)
Meal timing on the day of CA	
Fasting	59 (73.7)
Non‐fasting	21 (26.3)

*Note:* Data are expressed as the mean ± standard deviation or median and interquartile range, or number (%) of patients.

Abbreviations: AF atrial fibrillation; BMI body mass index; BNP brain natriuretic peptide; CA catheter ablation; DOAC direct oral anticoagulants; LA left atrium; LVEF left ventricular ejection fraction; TIA transient ischemic attack.

### 3.2 Comparison of Esophageal Images Using ICE and Preoperative CT

**Figure 4:**
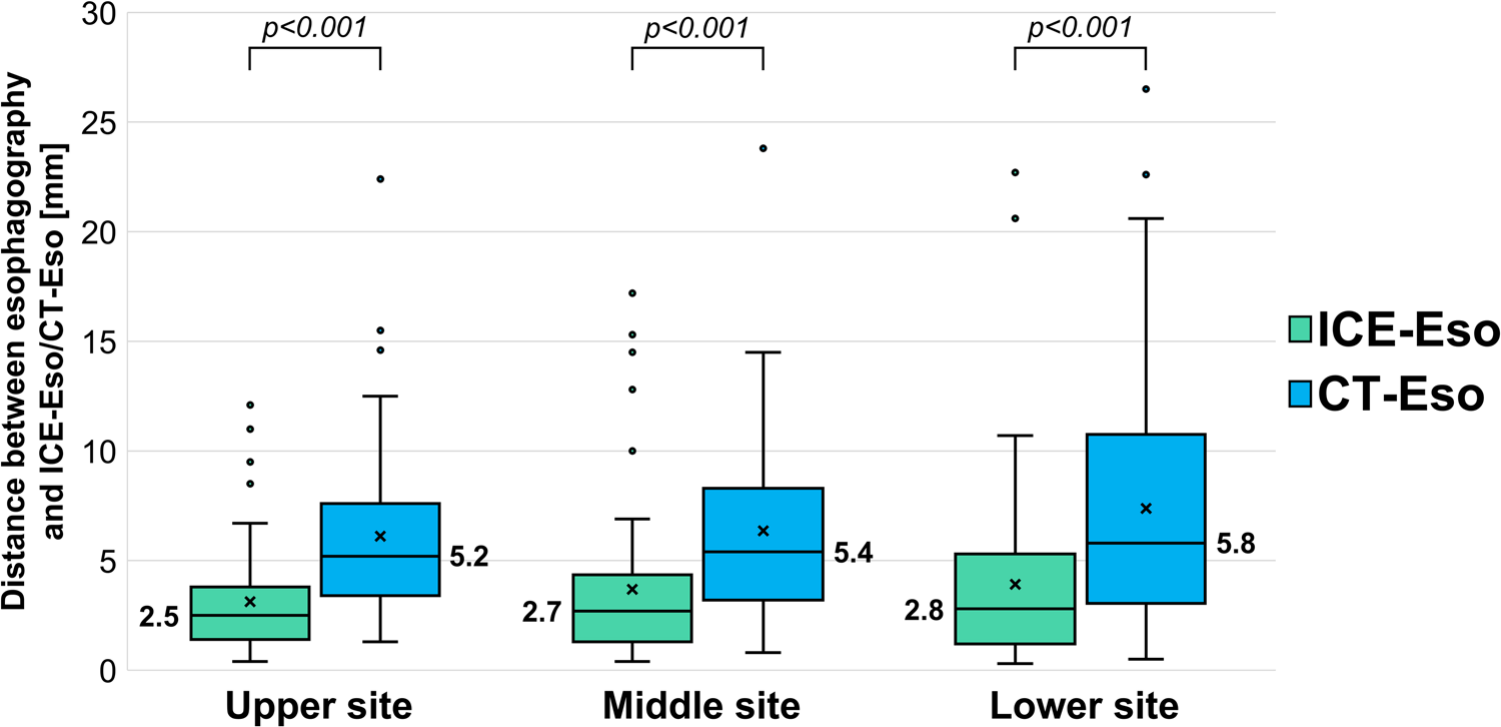
Distance between contrast esophagography and ICE‐Eso/CT‐Eso using the centerline of each image in three sections of the esophagus (upper, middle, and lower sites).

Seventy‐six patients (95%) successfully underwent ICE‐Eso; four patients (5%) were excluded due to difficulty in catheter manipulation. The mean number of slices acquired by ICE was 6.8 ± 3.4 slices, and the mean ICE‐Eso creation time was 172 ± 108 s. A significant difference was found between the two distances in all three sections: upper site (ICE‐Eso: 2.5 [1.4–3.6] mm vs. CT‐Eso: 5.2 [3.4–7.6] mm, *p* < 0.001), middle site (ICE‐Eso: 2.7 [1.3–4.3] mm vs. CT‐Eso: 5.4 [3.2–8.3] mm, *p* < 0.001), and lower site (ICE‐Eso: 2.8 [1.2–5.2] mm vs. CT‐Eso: 5.8 [3.1–10.3] mm, *p* < 0.001) ***(*** Figure 4 ***)***. Furthermore, we found that ICE‐Eso showed good matching with esophagography (67/76 patients, 88.1%) compared to CT‐Eso (41/80 patients, 51.2%) (*p* < 0.001). The Kruskal‐Wallis and post hoc tests demonstrated that the esophageal width was narrowest on esophagography compared to that of ICE‐Eso and CT‐Eso (esophagography: median [IQR]: 16.1 [14.0–18.8] mm, ICE‐Eso: 17.9 [14.7–20.4] mm, and CT‐Eso: 17.7 [15.9–21.8] mm; esophagography vs. ICE‐Eso, *p* = 0.065; esophagography vs. CT‐Eso, *p* = 0.0021, ICE‐Eso vs. CT‐Eso, *p* = 0.60).

### 3.3 HPSD Ablation Parameter Near the ICE‐Eso

We performed PVI in 75 patients (93.7%), because five (6.2%) had already undergone PVI in a previous procedure. The percentages of first‐pass PVI were 86.6% (65/75) and 85.3% (64/75) for the left and right PVs, respectively. Superior vena cava isolation and cavotricuspid isthmus ablation were performed in 23 (28.7%) and 55 (68.7%) patients, respectively. Non‐PV foci were documented in five patients (6.25%); therefore, additional RF deliveries were performed. In 66/80 patients (82.5%), the HPSD ablation was performed on the LAPW near the esophagus during the left PVI; this was visualized using ICE‐Eso. In two patients (2.5%), HPSD ablation was performed on the LAPW near the ICE‐Eso during the LAPW isolation. Among the remaining 12 patients (15%), 10 (12.5%) received AI‐guided PVI without HPSD ablation due to the distance from the PVI line; the other 2 did not require HPSD ablation because the prior PVI had been maintained. Analysis of HPSD ablation parameters near the esophageal location showed an RF application of 6.4 ± 2.8 times, a mean CF of 10.4 ± 2.4 g, a force‐time integral of 59 ± 13, and an AI of 284 ± 91. Table 2 details the procedural characteristics of HPSD ablation protocols.

**Table 2:** Details of ablation parameter for pulmonary vein isolation.

	*n* = 80
Left pulmonary vein	75 (93.7)
First‐pass isolation	65 (86.6)
LA‐PV epicardial connection	6 (8)
Right pulmonary vein	75 (93.7)
First‐pass isolation	64 (85.3)
LA‐PV epicardial connection	7 (9.3)
HPSD parameter on the ICE‐Eso	68 (87.1)
HPSD using left PVI line	66 (82.5)
HPSD using right PVI line	0 (0)
HPSD using LAPW isolation line	2 (2.5)
Number of RF application	6.8 ± 2.8
Total RF time using HPSD, s	36 ± 16
Contact force, g	10.4 ± 2.4
Impedance drops, ohm	7.7 ± 2.6
Force time integral	59.5 ± 13.3
Ablation index	284 ± 91
Acute gap site on LAPW using HPSD	5 (7.3)

*Note:* Data are expressed as the mean ± standard deviation or number (%) of patients.

Abbreviations: HPSD, high‐power short‐duration; LA, left atrium; LAPW, left atrium posterior wall; PV, pulmonary vein; PVI, pulmonary vein isolation; RF, radiofrequency.

The mean fluoroscopy time was 24.3 ± 7.5 min, and the mean procedure time was 152 ± 31 min. No severe complications, such as cardiac tamponade or stroke, were associated with the ablation procedure. One patient (1.2%) experienced gastroparesis with abdominal pain on the day after the procedure, which was diagnosed using abdominal CT. She maintained a fasted state for 14 days and subsequently recovered with oral mosapride (15 mg/day) without requiring endoscopy or barium examination. After 1 month of medical treatment, the abdominal pain resolved, and 3 months later an abdominal CT showed improvement in gastric distension *(*Supplemental Figure 2). After a mean follow‐up period of 728 (IQR, 545–882) days, 11 of the 80 (13.7%) patients developed atrial tachy‐arrythmias; 3 of the 11 patients underwent redo procedures, and PVI was achieved in all.

### 3.4 Predictors of Esophageal Movement Compared to Preoperative CT Imaging

Esophageal movement was defined as a deviation of > 5.0 mm in at least two of three sections between esophagography and CT‐Eso. The patients were divided into two groups: those with esophageal movement (Group A; *n* = 42) and those without (Group B; *n* = 38) (Table 3). We investigated the factors that affected esophageal movement, including whether the patient had fasted on the day of CA. Univariate analysis showed that esophageal movement was significantly higher in non‐fasting patients compared to those who had fasted (Group A: 16/42 patients [38%] vs. Group B: 5/38 patients [13.1%], *p* = 0.02). Moreover, subsequent multivariate analysis confirmed that non‐fasting on the day of CA correlated with esophageal movement compared to other predictive parameters. Univariate and multivariate logistic regression analyses of the predictors of esophageal movement are presented in Table 4.

**Table 3:** Comparison of patients with esophageal movement and those without esophageal movement.

	Esophageal movement (*n* = 42)	Non‐esophageal movement (*n* = 38)	*p* value
Age (years)	69.8 ± 9.4	71.2 ± 10.2	0.54
Gender (male)	30 (71.4)	23 (60.5)	0.34
BMI (kg/m^2^)	24.2 ± 4.5	24.5 ± 16.3	0.76
AF type			
Paroxysmal, *n*	17 (40.4)	19 (50)	0.50
Non‐paroxysmal, *n*	25 (59.5)	19 (50)	0.50
Hypertension, *n*	24 (57.1)	20 (52.6)	0.82
Diabetes, *n*	10 (23.8)	7 (18.4)	0.59
Congestive heart failure, *n*	11 (26.1)	13 (34.2)	0.47
History of stroke/TIA, *n*	6 (14.2)	2 (5.2)	0.26
CHA_2_DS_2_‐VASc score	2.6 ± 1.4	2.8 ± 1.2	0.62
LVEF, %	62.7 ± 8.6	58.3 ± 14.3	0.10
LA diameter, mm	42.7 ± 7.3	43.1 ± 8.3	0.80
LA volume index	40.8 ± 15	45.3 ± 17	0.24
BNP, pg/mL	120.0 [43.8–132.6]	180.5 [35.2–225.7]	0.18
Fasting on the day of CA	26 (62)	33 (86.8)	0.02

*Note:* Data are expressed as the mean ± standard deviation or median and interquartile range, or number (%) of patients.

Abbreviations: AF, atrial fibrillation; BMI, body mass index; BNP, brain natriuretic peptide; CA, catheter ablation; LA, left atrium; LVEF, left ventricular ejection fraction; TIA, transient ischemic attack.

**Table 4:** Univariate and multivariate logistic regression analyses of predictors for esophageal movement.

	Univariable analysis				Multivariable analysis			
Predictors	OR	95% CI		*p* value	OR	95% CI		*p* value
BNP	0.99	0.99	1.00	0.18	0.99	0.99	1.00	0.13
LVEF	1.03	0.99	1.07	0.10	1.02	0.98	1.07	0.26
Fasting on the day of CA	3.99	1.19	15.82	0.02	4.94	1.42	17.10	0.01

Abbreviations: BNP, brain natriuretic peptide; CA, catheter ablation; CI, confidence intervals; LVEF, left ventricular ejection fraction; OR, odds ratio.

## 4 Discussion

### 4.1 Major Findings

This study revealed two important findings. First, the matching rate between the ICE‐Eso and esophagography was higher than between the CT‐Eso and esophagography, and ICE‐Eso provided a more accurate real‐time esophageal location. We also determined that HPSD ablation using ICE‐Eso guidance was a safe and efficient method for PVI in the LAPW near the esophagus. Second, non‐fasting on the day of CA might have helped predict esophageal movement. Therefore, ICE‐Eso‐guided PVI was more useful for non‐fasting patients than for fasting patients.

### 4.2 Strategies for Avoiding Esophageal Injury

Consideration of strategies for avoiding esophageal injury is important. An esophageal temperature probe is a widely used, effective tool to avoid these complications.

A small study by Ishidoya et al. showed that a that shorter distance between the LA and the esophagus on preprocedural magnetic resonance imaging or CT could be a possible predictor of thermal esophageal injuries [18]. However, the esophagus has been reported to move even during CA; this is a critical consideration to prevent esophagus‐related complications during CA [19, 20]. One case report described an esophageal ulcer that developed after RF ablation of the right inferior PV, but in that patient, preprocedural CT showed that the esophagus was located on the left side [21].

A limitation of the present study was that, for cases in which the esophagus moved during CA, it was unclear whether the number of times the distance was measured before RF application contributed to image matching. However, the creation of multiple esophagus images during CA may complicate the procedure and extend its procedure time; therefore, we believe that creating images just once is an effective and simple method. Additionally, a previous study reported a high incidence of transmural injury in patients with right‐sided esophageal configurations (25%, 16%, and 7% in patients with right‐sided, central, and left‐sided configurations, respectively) [22].

Oikawa et al. reported that thermometer alarms were effective in preventing esophageal ulcers during RF ablation; however, their utility was limited in preventing gastric hypomotility during RF and cryoablation [23]. Yamane et al. reported on the usefulness of esophagograms in 2006; they manually injected approximately 10 mL of water‐soluble contrast medium was manually injected into the esophagus through the nasogastric tube for real‐time monitoring of the esophageal location behind the LA. They found that this technique helped to identify safe ablation zones as well as areas to avoid [24]. We believe that determining the esophageal location using ICE‐Eso is a useful method because gastrografin‐related complications, such as allergic reaction and lethal aspiration, have been reported [25, 26].

Pulsed‐field ablation (PFA) is a novel nonthermal ablation modality that differs from other ablation energy sources in its ability to preferentially ablate myocardial tissue. This unique feature supports its application in AF ablation, as it can decrease collateral damage, and the safety profile of PFA for the esophagus was confirmed [27, 28].

### 4.3 Predictor of Esophageal Movement During Catheter Ablation and Preoperative Fasting

Fasting is generally recommended before cardiac catheterization (e.g., percutaneous coronary interventions, implantation of cardiac implantable electronic device [CIED]). Several clinical studies have examined the effects of preoperative fasting on cardiac catheterization. An observational study that included over 1000 patients showed that the average fasting period was 11.6 h, which was much longer than recommended. The study also showed that longer periods of fasting increased the likelihood of patient discomfort [29]. A randomized trial from 2022 showed that a non‐fasting strategy was more beneficial for the well‐being of the patient and comparable to fasting in terms of safety for CIED procedures [30]. A study from 2024 demonstrated that non‐fasting was non‐inferior and superior to fasting when patients were under conscious sedation before cardiac catheterization, such as during coronary artery angiography, percutaneous coronary intervention, and CIED procedures [31]. Few other studies have reported on preoperative fasting and CA. The results of our study were different from those of previous studies in terms of the esophageal location during the CA procedure. Methods of sedation during CA vary greatly among facilities and countries [32]; therefore, it is difficult to conclusively verify that non‐fasting is always superior to fasting. However, when CA procedures were performed under conscious sedation, as they were at our hospital, non‐fasting was a potential factor in esophageal movement during CA. Therefore, this study suggests that fasting may be safer because it was associated with more accurate esophageal location during CA. Moreover, non‐fasting status before CA may pose a risk of aspiration under sedation. However, the safety implications require further verification, preferably through large‐scale clinical trials.

### 4.4 Limitations

This was a single‐center observational study that included a small number of patients.

One limitation was that endoscopic examinations were not performed due to the invasiveness of the procedure and the risk of COVID‐19 infection; therefore, the presence of asymptomatic lesions could not be ruled out. Future studies are necessary to examine the correlation between anatomical location and esophageal injury during RF ablation. Another limitation was that it was unknown whether our ICE‐guided esophageal visualization could help reduce gastric hypomotility, which cannot be detected using an esophageal temperature probe. The ability of ICE‐Eso to delineate esophageal anatomy more precisely and reduce such complications warrants further investigation with a larger number of patients. In addition, to our knowledge, no other clinical studies have compared fasting and non‐fasting before CA. Further clinical trials are needed to assess how they affect the well‐being and safety of the patient during CA.

## 5 Conclusions

This study demonstrated that ICE‐Eso was superior to CT‐Eso for determining real‐time, accurate esophageal location. Therefore, ICE‐Eso guided PVI for LAPW near the esophagus may reduce the risk of esophageal complications. Additionally, non‐fasting on the day of CA may help to predict esophageal movement.

## Ethics Statement

This study was approved by the Institutional Ethics Committee of the University of Yamanashi (approval no. 2773).

## Consent

The authors have nothing to report.

## Conflicts of Interest

The authors declare no conflicts of interest.

## Supporting information


**Supplemental Figure 1:** Pulmonary Vein Isolation Protocol for Atrial Fibrillation. Panels A and B: Final ablation lesion sets encircling the right and left pulmonary veins. Three‐dimensional mapping with the CARTOUNIVIU module of the left atrium (LA) in the posterior‐anterior (*left panel*) and left‐lateral (*right panel*) views. Red tags showed high‐power short‐duration applications at 50 W/5 s, and blue tags showed 40 W applications; targeted ablation index (AI) is 400 in the LA posterior wall non‐adjacent to the esophageal site, and 450 in the LA anterior wall.

**Supplemental Figure 2:** A Case of Gastric Hypomotility. Panel A: Abdominal radiograph showing gastric hypomotility (*yellow dotted line*). Panel B: Preoperative computed tomography (CT). Panel C: CT on postoperative day 2 showing gastric hypomotility (*yellow dotted line*). Panel D: Follow‐up CT three months after the procedure.

## Data Availability

The data that support the findings of this study are available on request from the corresponding author. The data are not publicly available due to privacy or ethical restrictions. The data that support the findings of this study are available from the corresponding author upon reasonable request.
