# Supplementary figures and images for: Visualization of Real‐Time Esophageal Location Using Intracardiac Echocardiography on a Three‐Dimensional Mapping System: Comparison of Esophageal Location Using Preoperative Computed Tomography and Investigation of Predictors for Esophageal Movement During Catheter Ablation
Source: J Cardiovasc Electrophysiol. 2025 Sep 19;36(11):3024–32. doi: 10.1111/jce.70096 (PMC12614146; doi:10.1111/jce.70096)

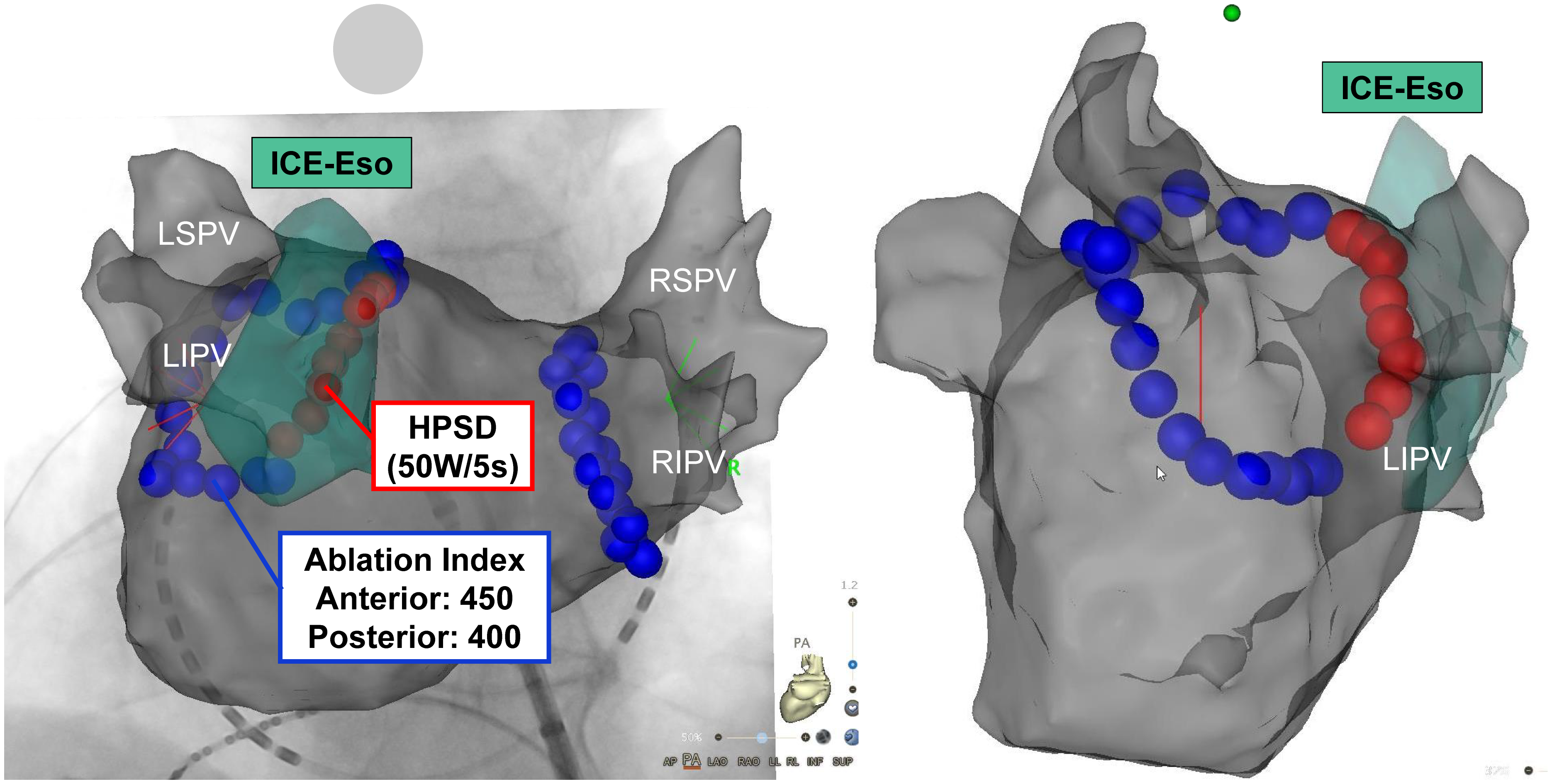

Supplement: Supplementary file 1 — Supplemental Figure 1: Pulmonary Vein Isolation Protocol for Atrial Fibrillation. Panels A and B: Final ablation lesion sets encircling the right and left pulmonary veins. Three‐dimensional mapping with the CARTOUNIVIU module of the left atrium (LA) in the posterior‐anterior (left panel) and left‐lateral (right panel) views. Red tags showed high‐power short‐duration applications at 50 W/5 s, and blue tags showed 40 W applications; targeted ablation index (AI) is 400 in the LA posterior wall non‐adjacent to the esophageal site, and 450 in the LA anterior wall. [file JCE-36-3024-s001.tif]

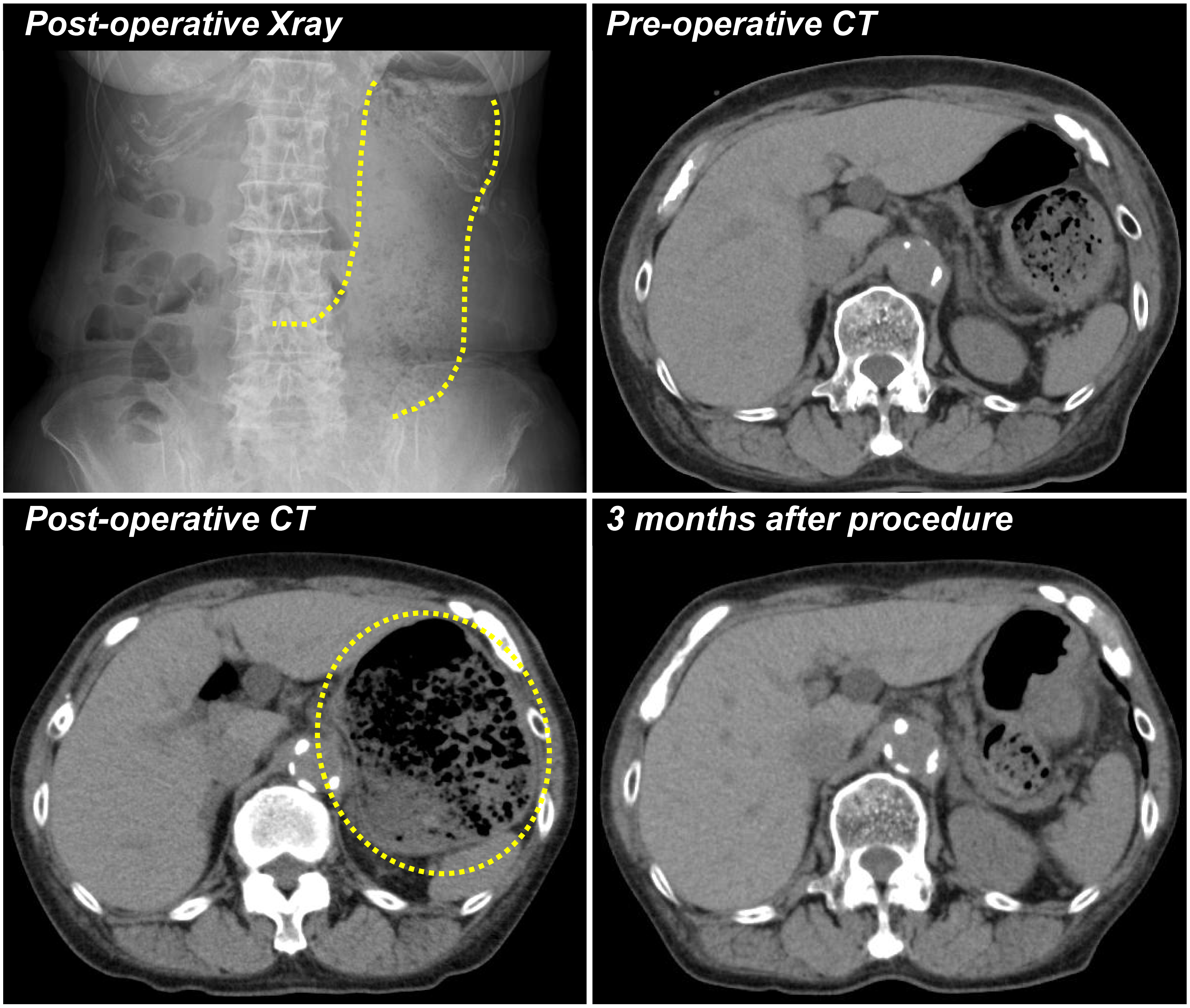

Supplement: Supplementary file 2 — Supplemental Figure 2: A Case of Gastric Hypomotility. Panel A: Abdominal radiograph showing gastric hypomotility (yellow dotted line). Panel B: Preoperative computed tomography (CT). Panel C: CT on postoperative day 2 showing gastric hypomotility (yellow dotted line). Panel D: Follow‐up CT three months after the procedure. [file JCE-36-3024-s002.tif]
